# Supplementary material for: A meta-analysis of sex differences in neonatal rodent ultrasonic vocalizations and the implication for the preclinical maternal immune activation model
Source: Biol Sex Differ. 2025 Jan 25;16:4. doi: 10.1186/s13293-025-00685-9 (PMC11762899; doi:10.1186/s13293-025-00685-9)
Supplement: Supplementary file 1 — Additional file 1. [file 13293_2025_685_MOESM1_ESM.docx]

**Supplementary Data**

**Baseline paper search terms**

**Google Scholar**

“pup ultrasonic vocalizations”|”neonate ultrasonic vocalization”|”maternal separation usv”|”isolation-induced ultrasonic vocalization”and “sex differences”|”sex”|”males”|”females”|"animal experimentation"  ==983 until June 2024

**PubMed**

"pup ultrasonic vocalization*" OR "isolation-induced ultrasonic vocalization*" OR "neonate ultrasonic vocalization*" OR "infant ultrasonic vocalization*" OR "maternal separation ultrasonic vocalization*" OR "neonate USV*" OR "pup USVs*" AND "sex differences*" == 22 papers

**MIA papers search terms**

**Google Scholar**

"maternal immune activation"|"MIA"|"poly ic"|"poly i:c"|"poly(i:c)"|"Polyinosinic:polycytidylic acid"|"lipopolysaccharide"|"lps"|"autism"|"autism spectrum disorder"|"asd"|"valproic acid" and "isolation-induced ultrasonic vocalizations"|"ultrasonic vocalization"|"usv"|"usvs"| and "sex"|"sex differences"|"male"|"males"|"female"|"females"|"animal experimentation" = 6600

**PubMed**

“maternal immune activation” OR “Polyinosinic:polycytidylic acid” OR “poly ic” OR “poly i:c” OR “lipopolysaccharide” OR “lps” “valproic acid” AND “isolation-induced ultrasonic vocalizations” OR “neonate ultrasonic vocalization” OR “pup usvs” AND “sex” = 62

*Note: The initial search was conducted on June 16, 2023 and a scan for new papers published in 2023 and 2024 was conducted on June 5, 2024.*


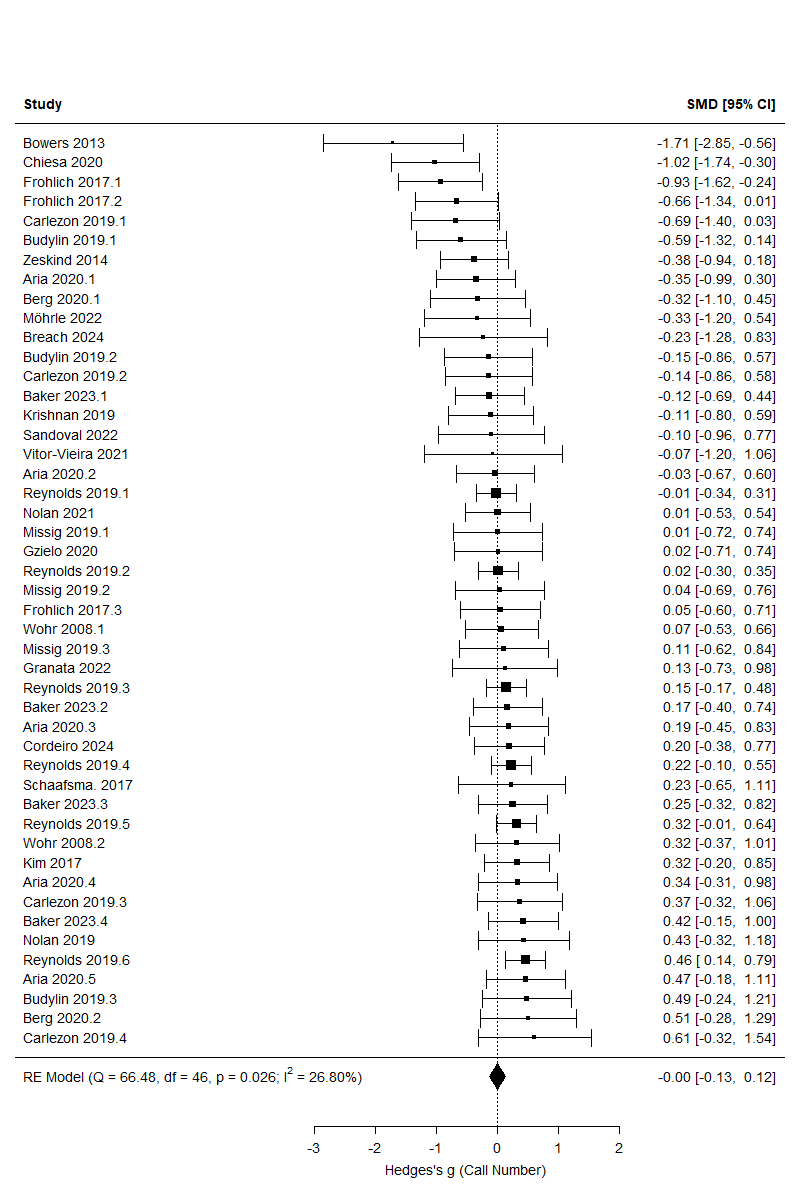


**Supplemental Figure 1.** Meta-analysis results of neonatal USV call number in response to brief maternal separation.


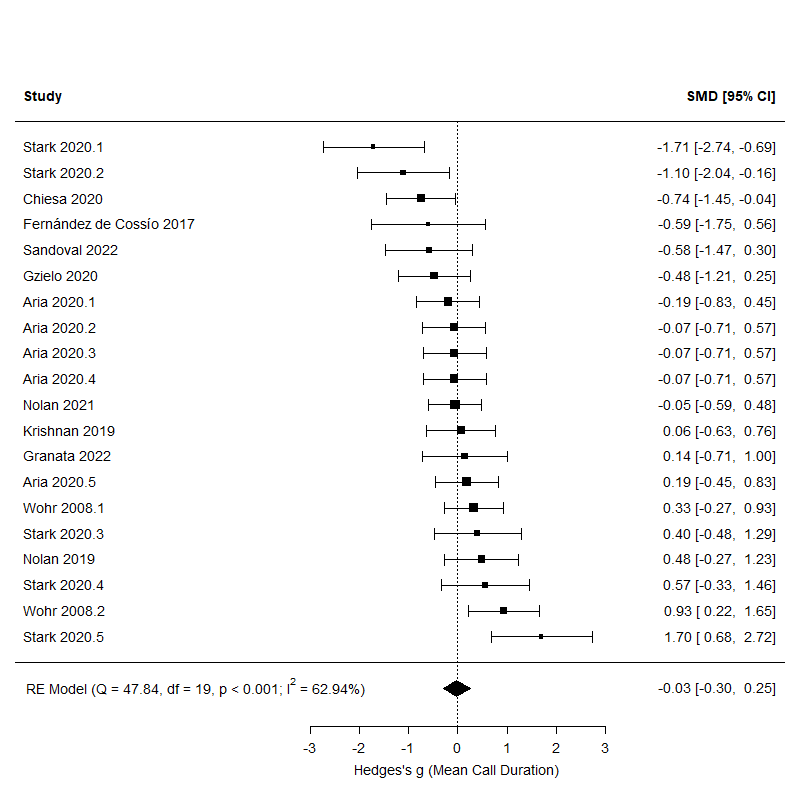


**Supplemental Figure 2.** Meta-analysis results of neonatal USV mean call duration in response to brief maternal separation.


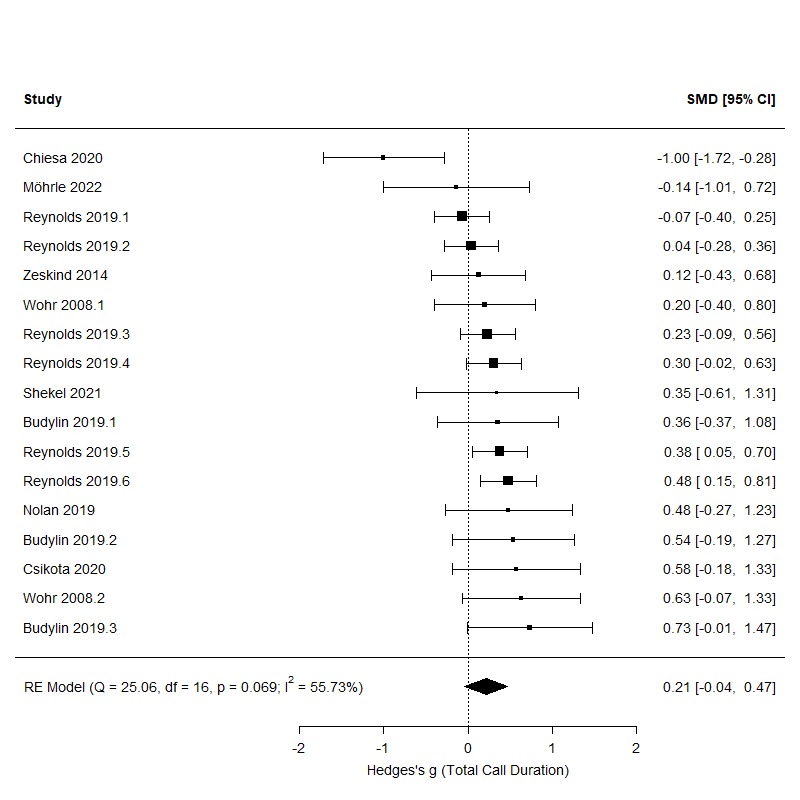


**Supplemental Figure 3.** Meta-analysis results of neonatal USV total call duration in response to brief maternal separation.


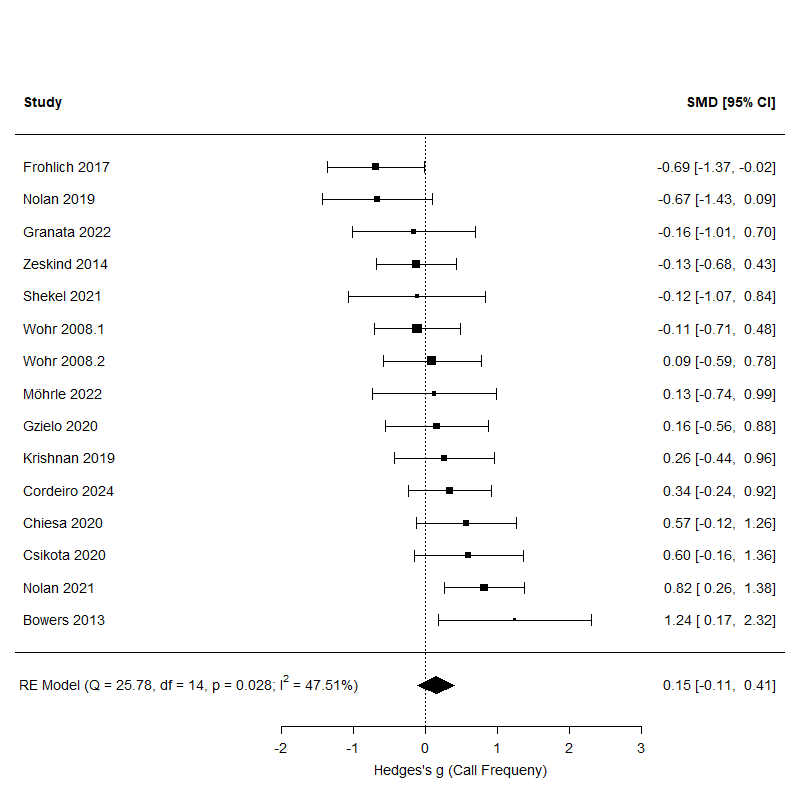


**Supplemental Figure 4.** Meta-analysis results of neonatal USV call frequency in response to brief maternal separation.


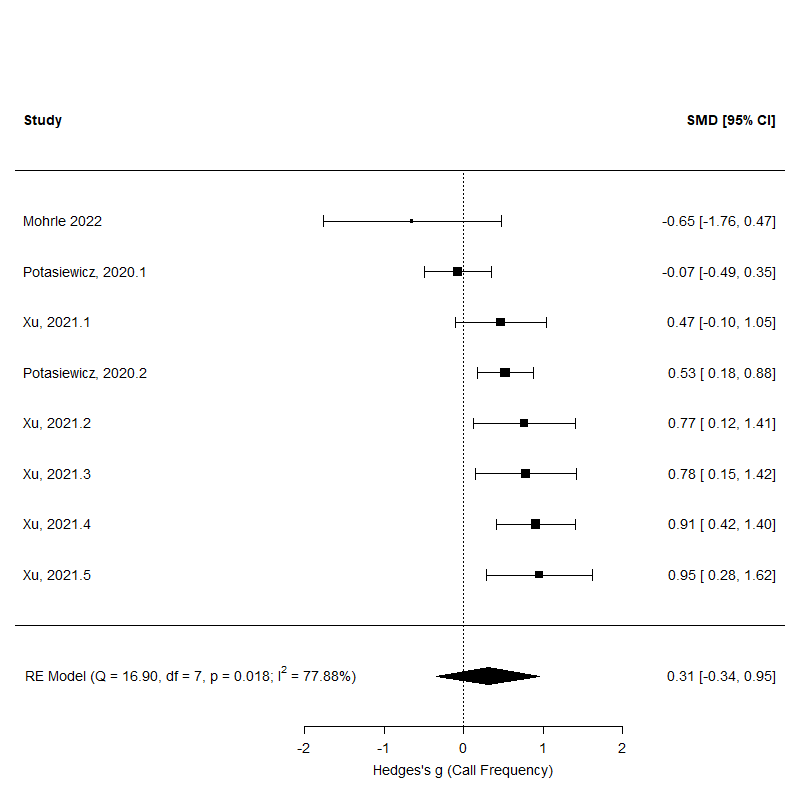
**Supplemental Figure 5.** Meta-analysis results of neonatal USV call frequency difference between control and maternal immune activation male rodents


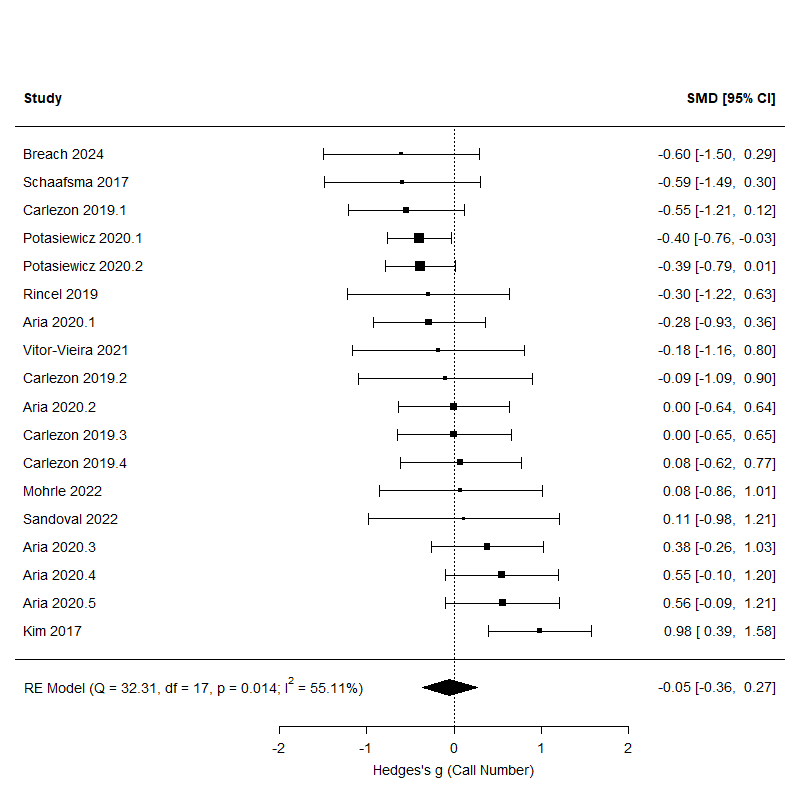


**Supplemental Figure 6.** Meta-analysis results of neonatal USV call number difference between control and maternal immune activation female rodents


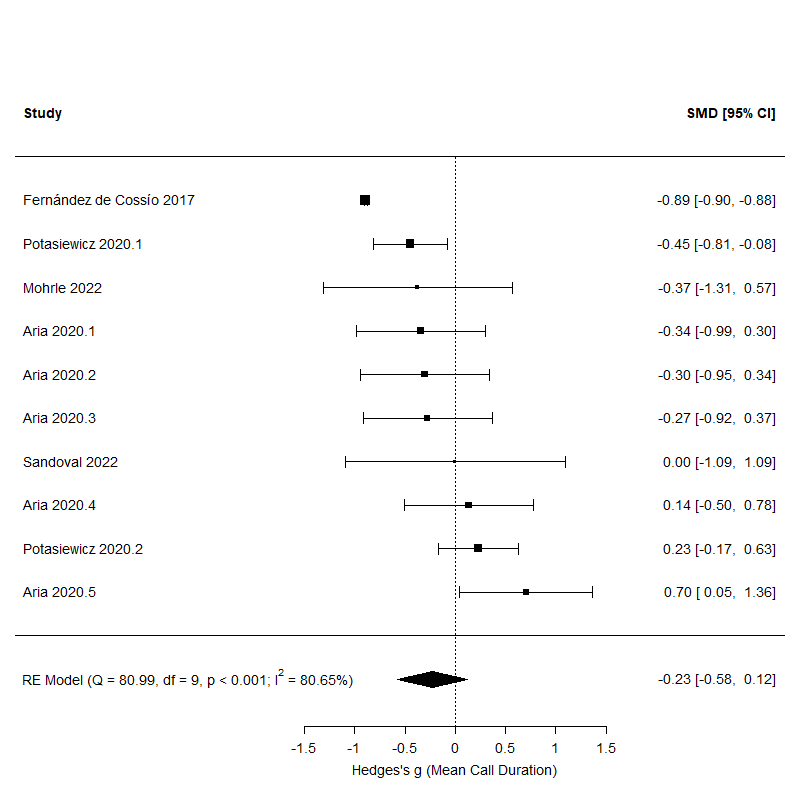


**Supplemental Figure 7.** Meta-analysis results of neonatal USV mean call duration difference between control and maternal immune activation female rodents


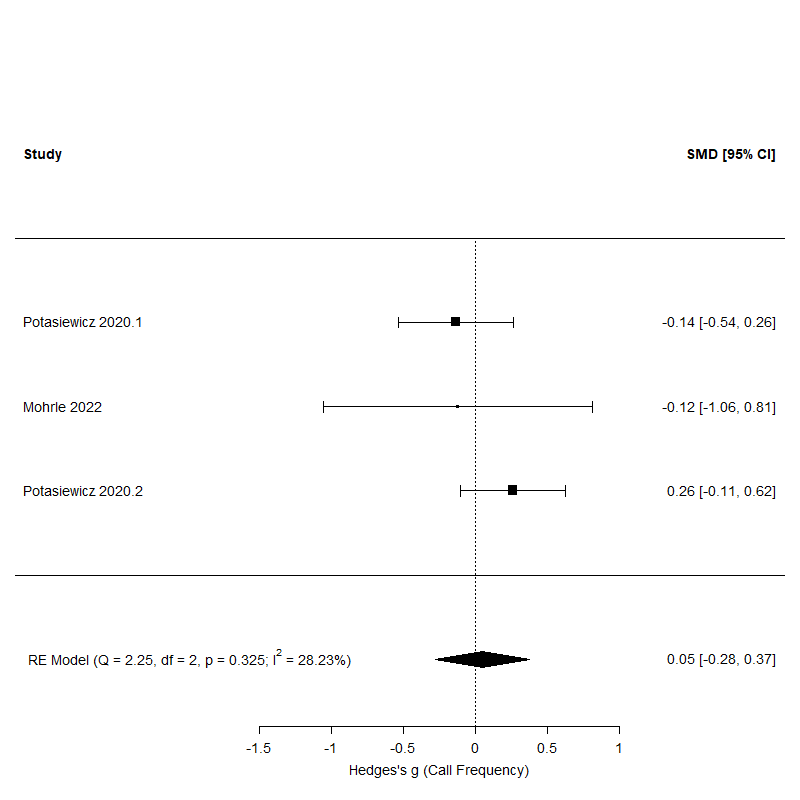
**Supplemental Figure 8.** Meta-analysis results of neonatal USV call frequency difference between control and maternal immune activation female rodents
